# Sustained intraocular VEGF neutralization results in retinal neurodegeneration in the Ins2^Akita^ diabetic mouse

**DOI:** 10.1038/srep18316

**Published:** 2015-12-16

**Authors:** Jose R. Hombrebueno, Imran HA. Ali, Heping Xu, Mei Chen

**Affiliations:** 1 Centre for Experimental Medicine, School of Medicine, Dentistry and Biomedical Sciences, Queen’s University Belfast, Belfast, UK

## Abstract

Current therapies that target vascular endothelial growth factor (VEGF) have become a mainstream therapy for the management of diabetic macular oedema. The treatment involves monthly repeated intravitreal injections of VEGF inhibitors. VEGF is an important growth factor for many retinal cells, including different types of neurons. In this study, we investigated the adverse effect of multiple intravitreal anti-VEGF injections (200 ng/μl/eye anti-mouse VEGF_164_, once every 2 weeks totalling 5–6 injections) to retinal neurons in Ins2^Akita^ diabetic mice. Funduscopic examination revealed the development of cotton wool spot-like lesions in anti-VEGF treated Ins2^Akita^ mice after 5 injections. Histological investigation showed focal swellings of retinal nerve fibres with neurofilament disruption. Furthermore, anti-VEGF-treated Ins2^Akita^ mice exhibited impaired electroretinographic responses, characterized by reduced scotopic a- and b-wave and oscillatory potentials. Immunofluorescent staining revealed impairment of photoreceptors, disruptions of synaptic structures and loss of amacrine and retinal ganglion cells in anti-VEGF treated Ins2^Akita^ mice. Anti-VEGF-treated WT mice also presented mild amacrine and ganglion cell death, but no overt abnormalities in photoreceptors and synaptic structures. At the vascular level, exacerbated albumin leakage was observed in anti-VEGF injected diabetic mice. Our results suggest that sustained intraocular VEGF neutralization induces retinal neurodegeneration and vascular damage in the diabetic eye.

Diabetic retinopathy (DR) affects almost 80% of individuals who have had diabetes for 1–2 decades^1^,^2^. Patients with DR may experience vision loss due to diabetic macular oedema (DMO) or proliferative DR (PDR) as a consequence of progressive retinal vascular dysfunction. DMO occurs when fluid leaks from damaged blood vessels into the macula, compromising central vision. According to the Wisconsin Epidemiologic Study of Diabetic Retinopathy, around 20% of type-1 and 14 ~ 25% of type-2 diabetic patients have DMO^1^,^3^. PDR (characterized by retinal neovascularization), is present in around 20% of diabetic patients who have suffered from the disease for 30 years^4^. Retinal laser photocoagulation has been used to treat DMO and PDR^5^,^6^. In addition, DMO has also been treated with intravitreal steroid injections^7^. Over the last few years, the intravitreal injection of neutralizing antibodies against vascular endothelial growth factor (VEGF) has become a mainstream therapy for DMO. The therapy has shown promising results in stabilising DMO and reducing retinal thickness^8^,^9^,^10^,^11^,^12^ as well as in improving visual acuity in PDR^13^,^14^,^15^.

VEGF is known to play a crucial role in the pathogenesis of DMO by promoting blood-retinal barrier (BRB) disruption and exacerbated vascular leakage^16^,^17^. Since the intravitreal injection of anti-VEGF antibody does not tackle the upstream fundamental factors that cause DMO, symptoms may re-occur once intraocular drug levels decline. Therefore, repeated injections (every 4 ~ 6 weeks) are needed to maintain normal BRB function in diabetic eyes. VEGF plays an important role in the survival and function of retinal cells, including neurons^18^,^19^. VEGF is involved in a wide range of neuronal functions, including neurogenesis, neuronal survival and synaptogenesis (reviewed in^19^,^20^). Accordingly, the neutralization of VEGF in rodent models has been shown to be detrimental for retinal neurons. A previous study has shown that systemic neutralization of VEGF in mice resulted in substantial neural retinal cell death^21^. Also, genetic deletion of retinal pigment epithelium (RPE)-derived VEGF in adult mice promoted rapid vision loss due to photoreceptor dysfunction^22^. Furthermore, chronic inhibition of VEGF in adult rats leads to retinal ganglion cell (RGCs) loss^23^.

Anti-VEGF therapy has been a mainstay treatment for patients with neovascular age-related macular degeneration (nAMD) for 8–10 years. Although the therapy reduces neovascular lesion and improves vision, there is a high incidence of retinal geographic atrophy characterised by RPE damage and photoreceptor cell death following the therapy^24^. These observations suggest that sustained VEGF depletion may pose severe adverse effects to the human retina. This is of particular concern to DR patients as 1) their retinas are more fragile than nAMD retina, and 2) they are generally a lot younger than nAMD patients and are expected to live for many more years and would thus require more anti-VEGF injections. It is therefore crucial to understand the long-term adverse effects of the therapy in patients with DR. In support of this concern, a recent study by Park *et al.*^25^ showed that intravitreal injection of a single dose of anti-VEGF resulted in increased inner retinal neuronal degeneration in diabetic rats. To elucidate this further, we investigated the long-term adverse effects of multiple intravitreal anti-VEGF injections in the Ins2^Akita^ mouse, a model of type 1-diabetes^26^.

## Results

### Multiple intravitreal injections of anti-VEGF induced cotton wool spot like-lesions in the Ins2^Akita^ mouse

**Figure 1:**
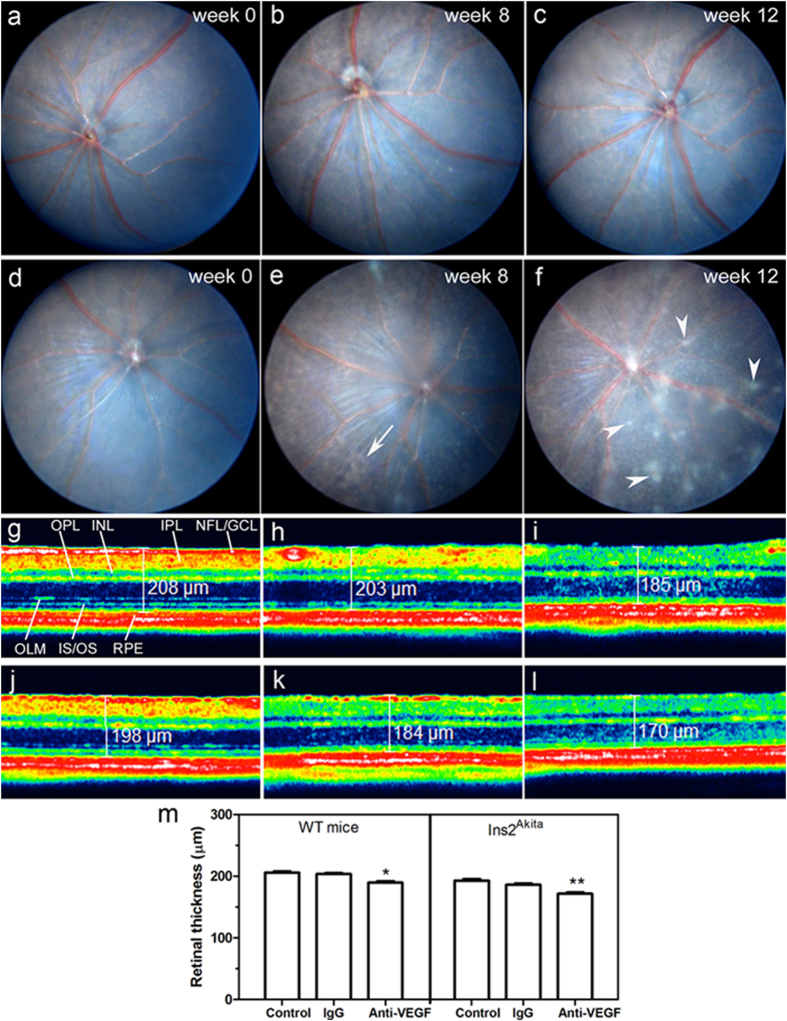
Clinical examinations following intravitreal injections of anti-VEGF. Fundus images from WT (**a–c**) or Ins2^Akita^ (**d–f**) mice at baseline (week 0) (a,d), 8 weeks (**b,e**) or 12 weeks (**c,f**) after intravitreal injections of anti-VEGF. *Arrows* indicate brownish irregular shaped lesions; *Arrowheads* indicate cotton wool spot-like lesions. SD-OCT representative images from WT (**g–i**) or Ins2^Akita^ (**j–l**) mouse retinas of non-injected controls (**g,j**), intravitreal IgG (**h,k**) or anti-VEGF (**i,l**) treated mice at 12 weeks post-injection. (**m**) Quantitative analysis of neuroretinal thickness in WT and Ins2^Akita^ non-injected, IgG or anti-VEGF treated mice (n = 6 eyes per strain/condition). Results are presented as mean ± SEM. *P < 0.05, **P < 0.01 compared to non-injected controls of the same strain. One-way ANOVA. NFL/GCL, nerve fibre/ganglion cell layer; IPL, inner plexiform layer; INL, inner nuclear layer; OPL, outer plexiform layer; OLM, outer limiting membrane; IS/OS, photoreceptor inner/outer segments; RPE, retinal pigment epithelium.

No fundus abnormalities were observed in WT and Ins2^Akita^ mice prior to intravitreal injections (Fig. 1a,d). Anti-VEGF treatment did not induce any retinal lesions in the WT mice (Fig. 1a–c). After 4 intravitreal injections of anti-VEGF antibody, 60% of the eyes developed patches of brownish irregular shaped lesions in the Ins2^Akita^ mouse (Fig. 1e). Following 5 injections, multiple whitish “cotton wool spot (CWS)”-like lesions were observed in 80% of the eyes in anti-VEGF treated Ins2^Akita^ mice (Fig. 1f). These whitish lesions were observed at the superficial layer (i.e. nerve fibre layer (NFL)) of the retina and on top of retinal vessels, and were spread in all retinal sectors varying in size from ¼ to 2 optic disc (OD) diameters. No retinal damage was detected in IgG treated WT and Ins2^Akita^ mice (Supplemental Fig. 1).

The thickness of the neuroretina was measured by Spectral Domain Optical Coherence Tomography (SD-OCT) (Fig. 1g–l). Anti-VEGF injections significantly reduced the neuronal retinal thickness in both WT and Ins2^Akita^ mice, although this reduction was more severe in Ins2^Akita^ mice (p < 0.01; Fig. 1g–m). Interestingly, the OCT-reflectance of the inner plexiform layer (IPL) and NFL/ganglion cell layer (GCL) was weaker in the anti-VEGF treated eyes (Fig. 1i,l). Intravitreal injection of IgG did not affect retinal thickness in WT and Ins2^Akita^ mice (Fig. 1m). Similar findings in fundus and SD-OCT images were observed when the experiment was repeated in a different set of mice.

### Retinal histopathological changes following multiple anti-VEGF injections

**Figure 2:**
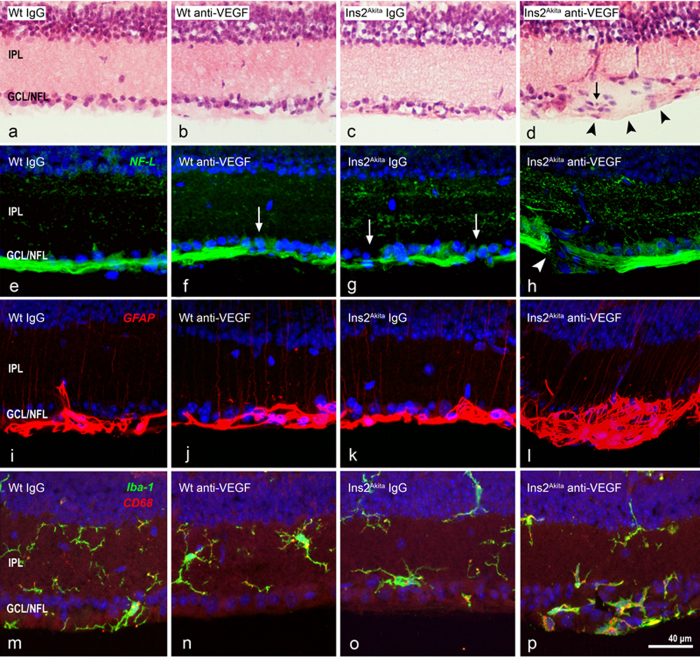
Histopathology of cotton wool spot (CWS)-like lesions after anti-VEGF treatment. Retinal sections from WT and Ins2^Akita^ mice after 5 intravitreal injections of IgG or anti-VEGF were processed for H&E staining (**a–d**) or NF-L (**e–h**), GFAP (**i–l**) and Iba-1/CD68 (**m–p**) immunoreactivities. (**d**) CWS-lesion at the GCL/NFL (*arrowheads*) accompanied by atypical cell infiltration (*arrow*). (**f,g**) Slight disruption of NF-L^+^ axonal fibres (*arrows*) in anti-VEGF treated WT (**f**) or IgG treated Ins2^Akita^ (**g**) mice. (**h**) Severe disruption of NF-L^+^ axonal fibres (*arrowhead*) in anti-VEGF treated Ins2^Akita^ mice. (**l,p**) Focal up-regulation of GFAP and infiltration of Iba-1^+^CD68^+^ microglial cells in CWS-lesions. IPL, inner plexiform layer; GCL/NFL, ganglion cell/nerve fibre layer.

Light microscopy analysis of hematoxylin and eosin (H&E) stained retinal sections showed no gross abnormalities in WT IgG, WT anti-VEGF or Ins2^Akita^ IgG treated mice (Fig. 2a–c). In contrast, Ins2^Akita^ mice injected with anti-VEGF exhibited focal lesions at the inner retinal layers, characterized by the swelling of the NFL/GCL and atypical cell infiltration (Fig. 2d, arrow). No morphological abnormalities were observed within the other layers of the retina. Immunostaining of retinal sections with NF-L showed normal distribution of RGC axons in IgG injected WT mice (Fig. 2e). Focal disruption or down-regulation of NF-L expression was observed in the retinas from anti-VEGF treated WT mice or IgG treated Ins2^Akita^ mice (Fig. 2f,g). Severe disruption or a total loss of focal NF-L expression was observed in retinas from Ins2^Akita^ mice following multiple anti-VEGF injections in focal areas corresponding to NFL/GCL swelling (Fig. 2h). Discrete mild NF-L disruptions, similar to that observed in Fig. 2f,g, were also detected in areas outside focal NFL/GCL swellings (data not shown). GFAP immunostaining revealed normal distribution of this intermediate filament in glial processes at the NFL/GCL and a faint thin expression at the IPL in IgG, anti-VEGF WT and IgG Ins2^Akita^ treated mice (Fig. 2i–k). Extensive focal GFAP up-regulation was observed in anti-VEGF treated Ins2^Akita^ mice (Fig. 2l). In addition, the accumulation of activated Iba-1^+^CD68^+^ microglial cells was observed at the NFL/GCL of anti-VEGF treated Ins2^Akita^ mice (Fig. 2p), in the areas corresponding to NFL/GCL swelling and focal GFAP up-regulation. Taken together, our results suggest that sustained VEGF neutralization to the retina of Ins2^Akita^ mice resulted in focal NFL damage, a classic pathological hallmark of CWS in human DR^27^,^28^.

### Electoretinogram responses following intravitreal anti-VEGF injection

**Figure 3:**
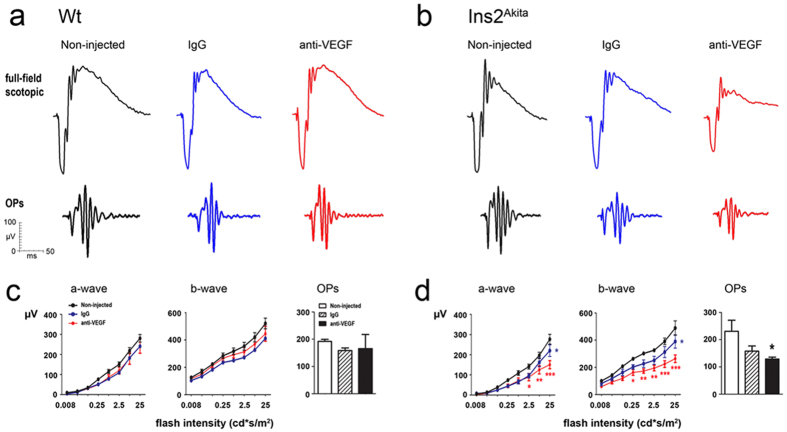
Abnormal electroretinogram (ERG) responses after anti-VEGF treatment. Scotopic ERG responses in WT and Ins2^Akita^ mice after 5 intravitreal injections of IgG or anti-VEGF. (**a,b**) Representative scotopic ERG responses from WT (**a**) and Ins2^Akita^ (**b**) mice of different treatment groups. (**c,d**) The amplitude (μV) of a-wave, b-wave and oscillatory potentials in WT (**c**) and Ins2^Akita^ (**d**) mice. (**c,d**) n = 5 mice per strain/condition. Results are presented as mean ± SEM. *P < 0.05, **P < 0.01, ***P < 0.001 compared to non-injected controls of the same strain. Two-way ANOVA.

No significant differences in scotopic electroretinogram (ERG) parameters were observed between WT non-injected controls and WT IgG or WT anti-VEGF treated groups (Fig. 3a,c). A slight but statistically significant reduction of both a- and b-wave amplitudes at the higher light intensity was observed in IgG treated Ins2^Akita^ mice (p < 0.05; Fig. 3b,d). Five intravitreal injections of anti-VEGF in the Ins2^Akita^ mouse resulted in a severe impairment of both a-wave and b-wave amplitudes and a significant reduction in the amplitudes of oscillatory potentials (OPs) compared to non-injected Ins2^Akita^ controls (p < 0.05; Fig. 3b,d).

### Retinal neuronal changes following multiple anti-VEGF intravitreal injections

#### Changes in photoreceptor cells

**Figure 4:**
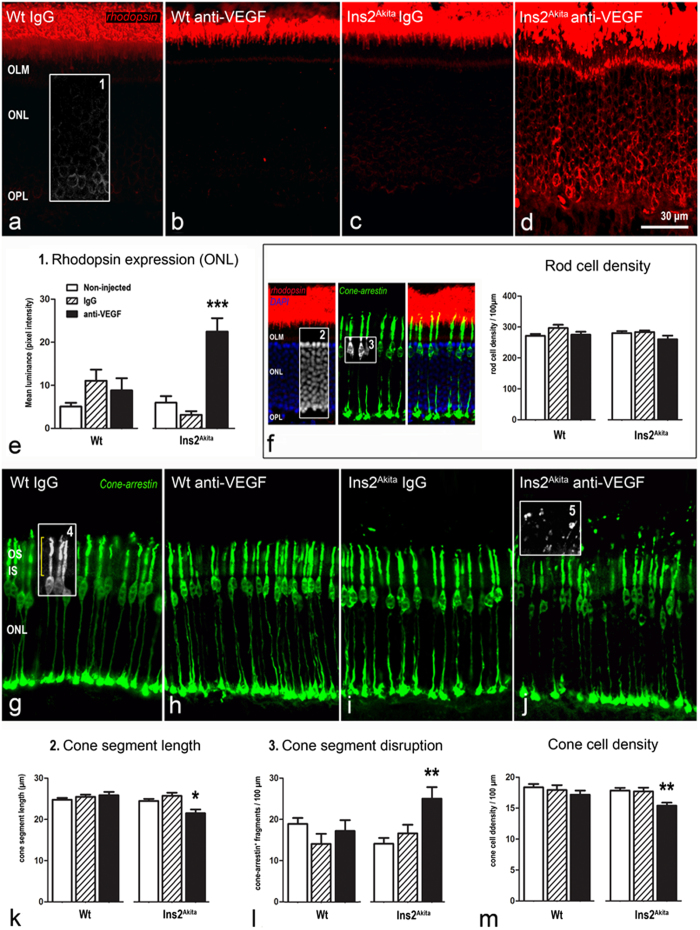
Photoreceptor abnormalities after anti-VEGF treatment. Retinal sections from WT and Ins2^Akita^ mice after 5 intravitreal injections of IgG or anti-VEGF processed for rhodopsin or cone-arrestin immunostaining. (**a–d**) Confocal images of rhodopsin immunoreactivity of different treatment groups. (**e,f**) Mean luminance values of rhodopsin immunostaining at the ONL (*box1*) and rod photoreceptor cell density (difference between DAPI^+^ nuclei (*box2*) and cone somata (*box3*) at the ONL). (**g–j**) Confocal images of cone-arrestin immunoreactivity of different treatment groups. (k-m) Quantitative analysis of cone segment length (*box4*) (**k**), cone segment disruption (cone-arrestin^+^ free particles above cone outer segments (*box5*)) (**l**) and cone photoreceptor density (**m**) in different treatment groups. n ≥ 20 retinal images per strain/condition. Results are presented as mean ± SEM. *P < 0.05, **P < 0.01, ***P < 0.001 compared to non-injected controls of the same strain. One-way ANOVA. OS, outer segments; IS, inner segments; OLM, outer limiting membrane: ONL, outer nuclear layer; OPL, outer plexiform layer.

Rod and cone photoreceptor cells were stained with antibodies against rhodopsin and cone-arrestin respectively (Fig. 4). Rhodopsin expression was confined to the outer segments of the rod photoreceptors in WT mice treated with IgG or anti-VEGF and in Ins2^Akita^ mice treated with IgG (Fig. 4a–c). Ectopical expression of rhodopsin within the inner segments and somata of rod photoreceptors was observed in anti-VEGF treated Ins2^Akita^ mice (Fig. 4d,e). No significant changes were observed in rod photoreceptor cell density within the different treatment groups (Fig. 4f).

Cone-arrestin staining showed that WT mice treated with IgG or anti-VEGF and Ins2^Akita^ treated with IgG had normal cone photoreceptor cell morphology (Fig. 4g–i). However, a reduction in the length of the cone photoreceptor segments and abnormal fragmentation were observed in anti-VEGF treated Ins2^Akita^ mice (Fig. 4j–l). Furthermore, cone photoreceptor cell density was also significantly reduced compared to non-injected controls (p < 0.01; Fig. 4m).

The thickness of the ONL was also significantly reduced in Ins2^Akita^ mice following anti-VEGF treatment. However, there was no reduction in ONL thickness in the IgG treated mice (Supplemental Fig. 2a,b).

#### Changes in outer plexiform layer and second order neurons

**Figure 5:**
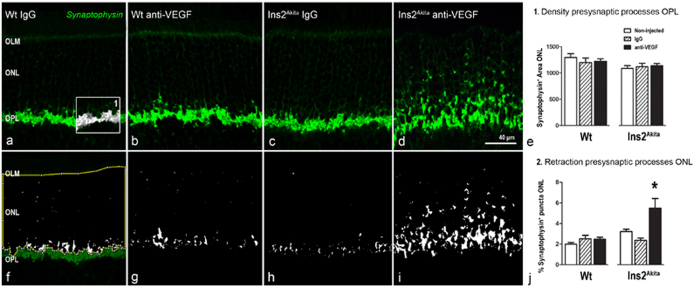
Pre-synaptic changes at the OPL after anti-VEGF treatment. Retinal sections from WT and Ins2^Akita^ mice after 5 intravitreal injections of IgG or anti-VEGF processed for synaptophysin immunoreactivity. (**a–d**) Confocal images of synaptophysin immunoreactivity from different treatment groups. (**e**) Quantitative analysis of synaptophysin^+^ area at the OPL (*box1*) and (**f**–**j**) ONL (thresholded white pixels enclosed in the yellow area (**f**)) in different treatment groups. n ≥ 20 retinal images per strain/condition. Results are presented as mean ± SEM. *P < 0.05 compared to non-injected controls of the same strain. One-way ANOVA. OLM, outer limiting membrane: ONL, outer nuclear layer; OPL, outer plexiform layer.

Presynaptic elements of rod and cone photoreceptor cells were investigated by using antibody against synaptophysin (Fig. 5). Synaptophysin was expressed predominately at the outer plexiform layer (OPL), although faint expression was also detected at the outer nuclear layer (ONL). There was no significant alteration in the distribution and expression level of synaptophysin in WT mice treated with IgG or anti-VEGF and in Ins2^Akita^ mice treated with IgG (Fig. 5a–h). In contrast, a significant increase in the expression of synaptophysin at the ONL was observed in anti-VEGF treated Ins2^Akita^ mice (p < 0.05; Fig. 5i,j), suggesting a marked withdrawal of rod terminals from the OPL.

**Figure 6:**
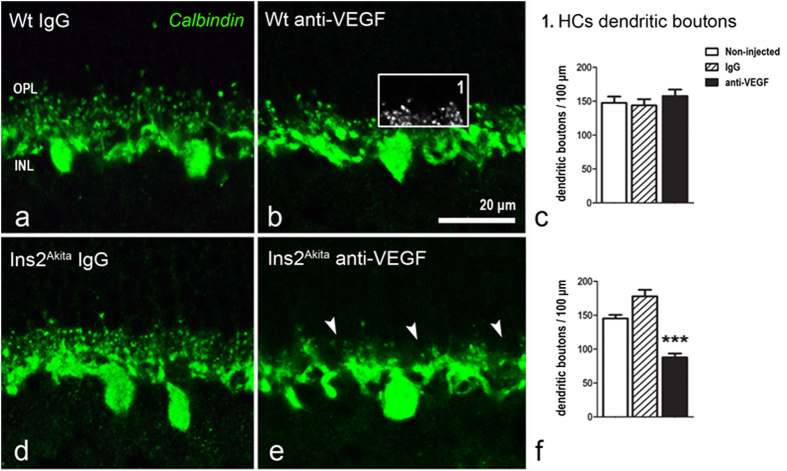
Horizontal cell dendritic boutons after anti-VEGF treatment. Retinal sections from WT (**a–c**) and Ins2^Akita^ mice (**d–f**) after 5 intravitreal injections of IgG or anti-VEGF processed for calbindin immunoreactivity. (**e**) Loss of horizontal cell dendritic boutons (*arrowheads*) at the OPL in anti-VEGF treated Ins2^Akita^ mice. The density of horizontal cell dendritic boutons (*box1*) in WT (**c**) and Ins2^Akita^ (**f**) mice. n ≥ 20 retinal images per strain/condition. Results are presented as mean ± SEM. ***P < 0.001 compared to non-injected controls of the same strain. One-way ANOVA. OPL, outer plexiform layer; INL, inner nuclear layer.

Synaptic elements of second order neurons were investigated by using antibodies against calbindin (horizontal cells), secretagogin (cone-bipolar cells) and PKCα (rod-bipolar cells). Neither intravitreal injections of IgG nor anti-VEGF affected the number and distribution of horizontal cell dendritic boutons in WT mice (Fig. 6a–c). However, a significant reduction of dendritic boutons was observed in the Ins2^Akita^ mouse following multiple anti-VEGF injections (p < 0.001; Fig. 6d–f). The density of horizontal-(data not shown), rod- and cone-bipolar cells (Supplemental Fig. 3) remained unchanged in WT and Ins2^Akita^ mice in all treatment groups. In addition, no significant changes were observed in the density of synaptic processes at the IPL (from either rod- or cone-bipolar cells) (Supplemental Fig. 3).

The thickness of the OPL was significantly reduced in Ins2^Akita^ mice following anti-VEGF treatment, in contrast to the IgG treated group, where the OPL thickness was unaffected (Supplemental Fig. 2c).

#### Changes in amacrine cells and retinal ganglion cells

**Figure 7:**
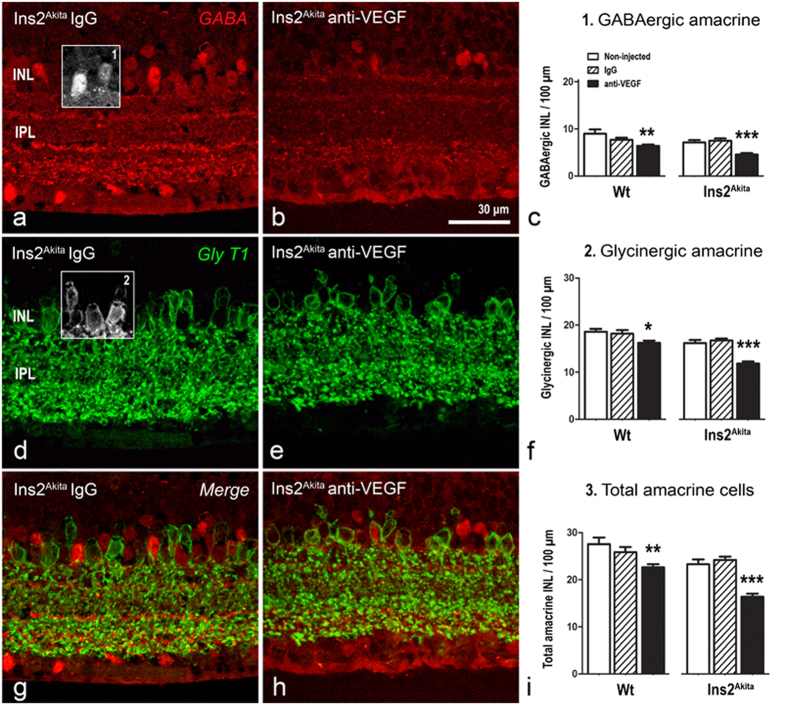
Amacrine cell loss in WT and Ins2^Akita^ anti-VEGF treated mice. Retinal sections from Ins2^Akita^ mice after 5 intravitreal injections of IgG or anti-VEGF processed for GABA (**a**,**b**) and GlyT1 (**d,e**) immunoreactivities. (**c,f,i**) The density of GABAergic (*box1*), glycinergic (*box2*) and total amacrine cells (GABAergic + glycinergic) at the INL in different treatment groups. n ≥ 20 retinal images per strain/condition. Results are presented as mean ± SEM. *P < 0.05, **P < 0.01, ***P < 0.001 compared to non-injected controls of the same strain. One-way ANOVA. INL, inner nuclear layer; IPL, inner nuclear layer.

GABAergic and glycinergic amacrine cells were evaluated using antibodies against GABA and GlyT1 respectively (Fig. 7). Compared to the non-injected controls, both WT and Ins2^Akita^ mice treated with anti-VEGF presented a significant reduction of GABAergic and glycinergic amacrine cells (p < 0.05; Fig. 7a–f). The total number of amacrine cells (GABAergic + glycinergic) was significantly reduced in both WT and Ins2^Akita^ mice following multiple anti-VEGF but not IgG injections (p < 0.01; Fig. 7g–i), although the level of reduction was more pronounced in Ins2^Akita^ mice (Fig. 7i). This was reflected by a significant reduction of the overall thickness of INL and IPL in diabetic mice (Supplemental Fig. 2d,e).

**Figure 8:**
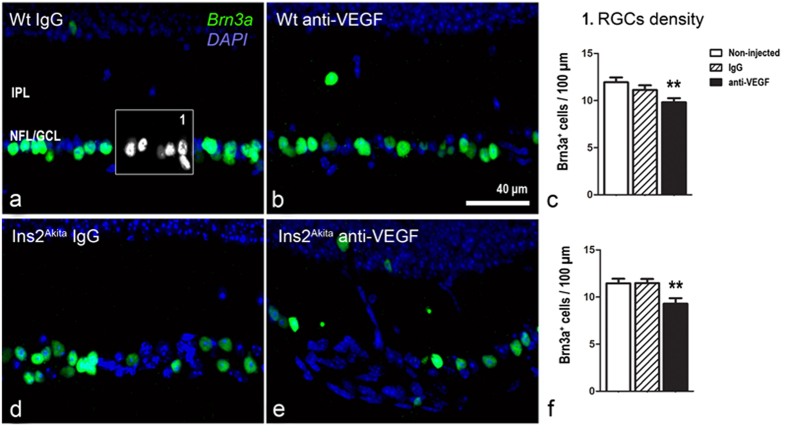
Retinal ganglion cell loss in WT and Ins2^Akita^ anti-VEGF treated mice. Retinal sections from WT (**a–c**) and Ins2^Akita^ mice (**d–f**) after 5 intravitreal injections of IgG or anti-VEGF processed for Brn3a immunoreactivity. (**c,f**) The density of Brn3a^+^ cells in different treatment groups. n ≥ 20 retinal images per strain/condition. Results are presented as mean ± SEM. **P < 0.01 compared to non-injected controls of the same strain. One-way ANOVA. IPL, inner nuclear layer; NFL/GCL, nerve fibre/ganglion cell layer.

Both WT and Ins2^Akita^ mice presented a significant drop in Brn3a^+^ RGCs following anti-VEGF injections compared to the non-injected controls (p < 0.01; Fig. 8). The injection of isotype IgG did not affect RGCs density in both WT and Ins2^Akita^ mice.

### Retinal vascular changes following multiple anti-VEGF intravitreal injections

**Figure 9:**
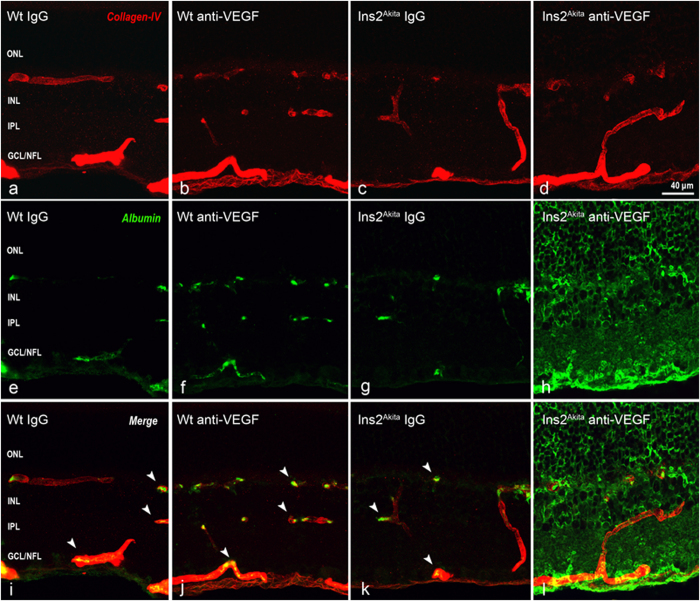
Retinal vascular changes in WT and Ins2^Akita^ anti-VEGF treated mice. Retinal photomicrographs of collagen-IV (red) and albumin (green) immunoreactivities in WT (**a,b,e,f,i-i**) and Ins2^Akita^ (**c,d,g,h,k,l**) after 5 intravitreal injections of IgG or anti-VEGF. (**i–k**) Albumin confined within retinal vessels (arrowheads). ONL, outer nuclear layer; INL, inner nuclear layer; IPL, inner nuclear layer; GCL/NFL, ganglion cell layer/nerve fibre layer.

Collagen IV immunostaining in WT mice showed normal distribution of the retinal vascular tree, characterised by three horizontal (ganglion cell layer (GCL), inner plexiform layer (IPL)/inner nuclear layer (INL) and OPL) and two interconnecting vertical plexuses (IPL and INL) (Fig. 9a–d). No changes were observed in the distribution and morphology of the different retinal vascular plexuses in WT and Ins2^Akita^ mice in all treatment groups (Fig. 9a–d). In WT mice treated with IgG or anti-VEGF and in Ins2^Akita^ mice treated with IgG, albumin immunostaining was mostly confined within retinal vessels (Fig. 9e–g,i–k, *arrowheads*). However, diabetic mice treated with anti-VEGF showed marked extravascular staining of albumin throughout all retinal layers (Fig. 9h,l), suggesting abnormal leakage from retinal vessels.

## Distribution of antibodies in mouse retina following intravitreal injection

To understand whether anti-VEGF antibody could diffuse into different retinal layers following intravitreal injection, fluorophore-conjugated anti-goat IgG was used to stain retinal sections from eyes receiving either anti-VEGF or IgG injections. Goat IgG immunoreactive puncta were detected in all retinal layers 24h following intravitreal injection of anti-VEGF or control IgG (Supplemental Fig. 4), suggesting that the antibodies were efficiently distributed through all retinal layers.

## Discussion

In this study, we showed that multiple intravitreal injections of anti-VEGF antibody resulted in significant neuroretinal degeneration and vascular leakage in Ins2^Akita^ diabetic mice. Although most of the retinal neurons were affected, those within the inner retina, such as amacrine cells and RGCs presented more severe degeneration than neuronal cells close to the outer retina. Sustained VEGF neutralization also affected the neuroretina of WT mice, although the damage was restricted to the inner retina and to a lesser degree compared to diabetic mice.

One of the most interesting observations of this study is the development of multiple CWS-like lesions in Ins2^Akita^ mice following ≥5 intravitreal anti-VEGF injections. CWS is often observed in the fundus of patients with diabetes or hypertension^28^,^29^, but it has never been reported in rodent models. It is believed that they may evolve as boundary sentinels of inner retinal infarction resulting from focal ischemia^27^,^28^. Focal ischemia may cause axonal damage and consequently the obstruction of axoplasmic transport and accumulation of axoplasmic material within the NFL, promoting the focal swelling of the retinal surface and disruption of neurofilament^27^. We also observed focal disruptions of neurofilament and accumulation of Iba-1^+^CD68^+^ microglial cells in the anti-VEGF treated Ins2^Akita^ mice. Infiltration of activated microglia has also been reported in human CWS^30^ as an adaptive immune response to axonal damage. The up-regulation of GFAP may reflect reactive gliosis from astrocytes and Müller cells due to neuronal damage^31^. The CWS was only observed in diabetic mice following 5 intravitreal anti-VEGF injections, suggesting the cumulative adverse effect of VEGF neutralization to inner retinal neurons. Whether NFL damage is caused by the direct effect of VEGF deprivation from neurons or secondary to vascular degeneration warrants further investigation.

Anti-VEGF mediated inner retinal damage was also characterized by the substantial reduction of amacrine cell and RGC densities. This was observed in both WT and Ins2^Akita^ mice, although the latter exhibited more severe damage and dysfunctional changes (evidenced by abnormal OPs responses). Amacrine and RGC degeneration has been reported in STZ-induced diabetic rats following a single intravitreal injection of anti-VEGF^25^. RGC loss has also been reported in the healthy rat retina following intravitreal injection of Bevacizumab^32^ or after chronic inhibition of VEGF-A^23^, although this was not confirmed by others^33^,^34^. Our results suggest that sustained VEGF neutralization is detrimental to inner retinal neurons not only under diabetic conditions, but also in the healthy retina.

The neuronal structure of the outer retina in diabetic mice was also affected by sustained VEGF neutralization. This was evidenced by a significant reduction in cone photoreceptor cell density, disruption of cone photoreceptor segments and redistribution of rhodopsin in rod cell somata (all well-known signs of photoreceptor cell stress^35^,^36^,^37^), and synaptic impairment at the OPL. The cellular changes in photoreceptor cells were in concordance with the abnormal scotopic a-wave response observed in the ERG analysis. VEGF is an important survival factor for photoreceptors and systemic neutralization of VEGF results in photoreceptor cell apoptosis^21^. In addition, VEGF-receptor 2, known to mediate pro-survival signals in neuronal cells^19^,^23^ is strongly expressed in photoreceptor cells^21^. VEGF is also critically involved in synaptic plasticity and function^19^,^38^. Cone photoreceptor cell loss and synaptic impairment of the OPL features retinal neurodegeneration in rodent models of DR^39^,^40^, including the Ins2^Akita^ mouse^41^. Our results suggest that sustained VEGF neutralization may accelerate outer retinal degeneration in diabetic Ins2^Akita^ eyes. This may be of significant relevance, as human DR also presents outer retinal deficits^42^,^43^. In addition to the direct effect of VEGF depletion on neurons, other factors, in particular anti-VEGF associated vascular damage may also contribute to retinal neuronal degeneration (see below) as chronic VEGF antagonism is known to affect the integrity of the choriocapillaris^22^.

Although both inner and outer retinal neurons were affected by intravitreal anti-VEGF injections, the damage in the inner retina was more severe than that observed in the outer retina. The antibody can efficiently diffuse to all retinal layers within 24h after injection, suggesting that the increased damage in the inner retina (i.e., RGCs and amacrine cells) is not caused by unequal distribution of the antibody, rather, it may suggest that inner retinal neurons are more sensitive to VEGF depletion. At least two factors may contribute to this phenomenon. Firstly, inner retinal neurons may be more susceptible to hyperglycaemia than outer retinal neurons and therefore more fragile to changes in the availability of neurotrophic factors. In this regard, ganglion cell and amacrine cell degeneration has been frequently observed in diabetic experimental models^41^,^44^. Secondly, the inner BRB damage caused by VEGF depletion may result in vascular dysfunction-dependent neurodegeneration.

VEGF is an important growth factor for post-mitotic neurons in the central nervous system^19^,^23^ and vascular endothelial cells^45^. Under diabetic conditions, retinal neurons, endothelial cells and pericytes undergo progressive degeneration^46^ as a result of sustained hyperglycaemia. We found that repeated injection of anti-VEGF induced marked albumin leakage in the retina of diabetic mice - a sign of BRB disruption. Although retinal leukostasis and acellular capillary has been described in the Ins2^Akita^ mice, the mice do not develop severe DR related vasculopathy by 5 months of age^47^,^48^. Furthermore, the Ins2^Akita^ mice do not secrete abnormal levels of VEGF at this stage of DR^47^. Therefore, the lack of pathogenic retinal VEGF levels and/or severe VEGF-associated vasculopathy in Ins2^Akita^ mice may result in anti-VEGF antibody binding to “innocent” targets, including vascular cells and neurons, leading to further neuronal and vascular damage. The leakage of albumin and other plasma proteins into retinal parenchyma will inevitably induce inflammation, which may also contribute to retinal neuronal degeneration in anti-VEGF treated Ins2^Akita^ mice.

A number of factors may limit the translation of our results to clinical practice in DMO patients. Firstly, the diabetic mice only develop mild vascular degeneration, and may not have pathological VEGF in the retina. In this regard, the anti-VEGF treatment thought to neutralize retinal pathogenic levels of VEGF in diabetic patients may deplete physiological VEGF in diabetic mice. Secondly, the differences in the affinity of the anti-mouse VEGF antibody used in this study compared to those specific clinical formulations (e.g. Bevacizumab or Ranibizumab) make it difficult to predict to what level the VEGF neutralization is harmful to the diabetic retina. Moreover, other experimental factors such as cross-species reactivity (e.g. goat IgG to mice) and inflammatory reaction to buffers used in antibody stabilization may contribute to the retinal phenotype observed in anti-VEGF treated mice. Nevertheless, our results show that sustained neutralization of VEGF in a pre-clinical model of type-1 diabetes promoted retinal neurodegeneration and induced vascular leakage. Although the proven benefit of anti-VEGF therapy in most DMO patients, a significant number of patients do not respond to the therapy^49^,^50^, suggesting that other factors may be fundamental in the pathogenesis of DMO in those non-responders. If this is indeed the case, our results suggest that the anti-VEGF therapy may potentially carry an “off-target” risk and the therapy may need to be applied more judiciously to avoid non-desirable side-effects. Even for the responders, the frequency of anti-VEGF needs to be carefully controlled to avoid potential depletion of physiological VEGF. Further clinical studies on how anti-VEGF therapy may impact on retinal physiological VEGF levels, will help to understand whether the therapy carries any long-term risk to DMO patients.

## Methods

### Animals

Male heterozygous Ins2^Akita^ mice (3 months of age) of C57BL/6J background (Cat 003548 - originally purchased from Jackson Laboratory, Bar Harbor, USA) and age-matched non-diabetic siblings were used. The Ins2^Akita^ mice develop robust hyperglycemia by 4.5 weeks of age^26^. Ins2^Akita^ heterozygous males were bred with female C57BL/6J mice and diabetes confirmed by blood glucose test (>250 mg/dl) in 6 weeks old littermates. All mice were housed in a pathogen-free animal housing room on a 12/12-hour light/dark cycle with free access to food and water. All procedures were conducted under the regulation of the UK Home Office Animals (Scientific Procedures) Act 1986 and approved by the Ethical Review Committee of Queen’s University Belfast. The study was conducted in compliance with the Association for Research in Vision & Ophthalmology Statement for the Use of Animals in Ophthalmology and Vision Research.

### Intravitreal injection

Intravitreal injections were performed as previously described^51^. 1 μl of goat anti-mouseVEGF_164_ (200 ng/μl; endotoxin-free, AF-493-NA; R&D Systems, Abingdon, UK) or endotoxin-free goat IgG (200 ng/μl; AB-108C; R&D Systems) in 0.01 M phosphate buffered saline (PBS, pH 7.4) was injected in 3-month old Ins2^Akita^ or C57BL/6J siblings using a repeating dispenser (PB-600-1; Hamilton Bonaduz AG, Bonaduz, Switzerland). The AF-493-NA antibody has been shown to efficiently neutralize the bio-activity of mouse VEGF_164_ *in vivo*^52^. The dose for intravitreal injection was chosen from a previous study by Nishijima *et al.*^23^. The injections were performed in the right eye with a 2-week time interval. The experiment was conducted twice. The total number of injections was 6 in study 1 (n =6 mice per group) and 5 in study 2 (n = 6 mice per group). Age-matched mice that did not receive any intravitreal injections served as full controls in each study.

### Clinical investigations

Animals were anesthetized by isoflurane inhalation and pupils dilated using 1% tropicamide and 2.5% phenylephrine (Chauvin, Essex, UK). A topic endoscopic fundus imaging system was used as previously described^53^,^54^, to obtain fundus images at baseline level (prior the first injection) and 2 weeks following each intravitreal injection. For SD-OCT examination, animals were anesthetized with ketamine hydrochloride (60 mg/kg; Fort George Animal Centre, Southampton, UK) and xylazine (5 mg/kg; Pharmacia & Veterinary Products, Kiel, Germany) and pupils dilated. The examination was conducted 2 weeks after the final intravitreal injection (n = 5 ~ 6 animals per group) using the Spectralis Heidelberg OCT system (Heidelberg Engineering, Heidelberg, Germany), set to a 30° field of view. The thickness of the neuroretina (from the NFL to the apical border of the RPE) was measured at ~600 μm distance from the optic disc (OD) in dorsal-ventral and nasal-temporal sectors by two independent researchers. Retinal thickness was then averaged in each mouse for further statistical analysis.

### Electroretinography

Scotopic ERGs responses were evaluated 2 weeks following the final intravitreal injection (n ≥ 5 animals per group) as previously described^41^. Briefly, dark-adapted mice were deeply anesthetized with ketamine hydrochloride and xylazine and pupils dilated. ERGs were recorded using mouse corneal ERG electrodes in response to single white light flash, delivered by a standard Ganzfeld Stimulator (LKC Technologies, Gaithersburg, MD, USA). The amplitude of the scotopic a-wave, b-wave and OPs (summed amplitude of wavelets 2–5) was measured.

### Immunohistochemistry

Two weeks following the final intravitreal injection with anti-VEGF or IgG, mice were sacrificed by CO_2_ inhalation and eyes dissected and fixed in 2% paraformaldehyde for 2h. Eyes were then processed for immunohistochemistry as previously described^55^ and examined by confocal microscopy (C1-Nikon Confocal Microscope, Eclipse TE200-U, Nikon UK Ltd, Surrey, UK). A list with the primary antibodies used in the study is shown in Supplemental Table 1. Some of the retinal sections were processed for standard H&E staining and examined by light microscopy (Nikon Eclipse E400- Nikon UK Ltd). The diffusion efficiency of anti-VEGF and control IgG antibodies was tested as previously reported by Michael *et al.*^56^ In brief, retinal cryosections from WT mice were stained with Alexa fluor-488 donkey anti-goat IgG for 2 h in room temperature (1:200) 24 hours after intravitreal injection with goat IgG or anti-VEGF.

### Retinal morphometry

Confocal images were used to quantify retinal neurons and synaptic structures (n = 3 animals per group). Data was obtained from 3 sections per eye at center-middle retinal eccentricities (0.6 and 1.5 from the OD). During image acquisition, confocal settings remained constant and images were then analyzed using FIJI software (National Institutes of Health, Bethesda, MD). The following parameters were analyzed: 1) rod and cone photoreceptor cell density (rods calculated as difference between the number of cone somata and DAPI^+^ nuclei at the ONL), 2) cone segment length (outer segment plus inner segment), 3) cone segment fragmentation (cone-arrestin^+^ free particles at the photoreceptor segment layer), 4) intensity of rhodopsin immunostaining at the ONL, 5) Area of synaptophysin-immunoreactive puncta at the OPL and ONL, 6) horizontal cell dendritic spine density, 7) horizontal cell, rod-bipolar and cone-bipolar cell density, 8) rod- and cone-bipolar cell axon bouton density, 9) GABAergic and glycinergic amacrine cell density, and 10) RGCs density. The thickness of each retinal layer (ONL, OPL, INL, IPL and GCL/NFL) was measured in cryosections stained for GABA, GlyT1 and DAPI by two independent researchers (Supplemental Fig. 2).

At least 20 retinal images per strain/age were analyzed and values averaged and normalized to 100 μm retinal length. The intensity of rhodopsin immunostaining was measured from confocal images captured under constant photomultiplier settings, in which the mean luminance values (average brightness per pixel) were calculated from manually traced areas across the ONL (from the OPL to the outer limiting membrane). Luminance values were estimated from an average selection of at least 1000 pixels to ensure a high degree of variance. Background images were acquired from a vacant area of the labeled section and subtracted from the raw images to eliminate background noise. To quantify the density of synaptophysin-immunoreactive puncta, the ONL was manually traced and inverted. Mean luminance values of synaptophysin immunostaining at the OPL were then used to define minimum threshold values and the resulting images analyzed via the FIJI *analyze particles* tool. The density of synaptophysin-immunoreactive puncta was normalized to the ONL area.

### Data analysis

SD-OCT retinal thickness and morphometric data in each strain of mice were analyzed using one-way ANOVA, followed by post-hoc Bonferroni’s pairwise comparisons. ERG responses (scotopic a-wave, b-wave and OPs) were analyzed using 2-way ANOVA followed by post-hoc Bonferroni’s pairwise comparisons. Data were expressed as mean ± SEM and p < 0.05 was considered statistically significant.

## Additional Information

**How to cite this article**: Hombrebueno, J. R. *et al.* Sustained intraocular VEGF neutralization results in retinal neurodegeneration in the Ins2^Akita^ diabetic mouse. *Sci. Rep.* **5**, 18316; doi: 10.1038/srep18316 (2015).

## Supplementary Material

Supplementary Information
