# Supplementary material for: Sustained intraocular VEGF neutralization results in retinal neurodegeneration in the Ins2Akita diabetic mouse
Source: Sci Rep. 2015 Dec 16;5:18316. doi: 10.1038/srep18316 (PMC4680939; doi:10.1038/srep18316)

**Sustained intraocular VEGF neutralization results in retinal neurodegeneration in the Ins2^Akita^ diabetic mouse**

Jose R. Hombrebueno, Imran HA. Ali, Heping Xu, Mei Chen

Centre for Experimental Medicine, School of Medicine, Dentistry and Biomedical Sciences, Queen’s University Belfast, Belfast, UK

**Primary antibodies used in the study**

| **Antigen** | **Antiserum** | **Dilution** | **Source** | **Retinal localization** |
| --- | --- | --- | --- | --- |
| Light neurofilament 68 KDa (NF-L) | rabbit anti-NF-L | 1:200 | Abcam | Horizontal and ganglion cell processes |
| Glial fibrillary acidic protein (GFAP) | rabbit anti-GFAP | 1:200 | Abcam | Müller cells and astrocytes |
| Iba-1 | rabbit anti-Iba1 | 1:400 | Wako | Microglial cells/retinal macrophages |
| CD68 | rat anti-CD68 | 1:200 | AbD Serotec | Activated microglia/macrophages |
| Rhodopsin | mouse-rhodopsin, clone 4D2 | 1:500 | Chemicon | Rod photoreceptors |
| Cone arrestin | rabbit anti-cone arrestin | 1:10000 | Chemicon | Cone photoreceptors |
| Synaptophysin | rabbit anti-synaptophysin | 1:200 | Dako | Presynaptic processes |
| Secretagogin | sheep anti-secretagogin | 1:400 | Biovendor R&D | Cone bipolar cells |
| PKCα | rabbit anti-PKCα | 1:500 | Santa Cruz | Rod bipolar cells |
| Calbindin | rabbit anti-calbindin | 1:1000 | Chemicon | Horizontal cells |
| γ-aminobutyric acid (GABA) | guinea pig anti-GABA | 1:500 | Chemicon | GABAergic amacrine cells |
| Glycine Transporter-1 (GlyT1) | goat anti-glyT1 | 1:3000 | Chemicon | Glycinergic amacrine cells |
| Brn3a | goat anti-Brn3a | 1:500 | Santa Cruz | Ganglion cells |
| Collagen-IV | rabbit anti-collagen IV | 1:200 | ABD Serotec | Retinal vessels |
| Albumin | goat anti-mouse albumin | 1:200 | Bethyl | Albumin |

**Supplementary figure 1**. **Fundus images of WT and Ins2^Akita^ mice following intravitreal injection of goat IgG.** Fundus images were taken from WT (a, b) and Ins2^Akita^ (c, d) mice at week 0 (immediately before intravitreal IgG injection) (a, c) and 12 weeks after intravitreal injections of IgG (b, d).


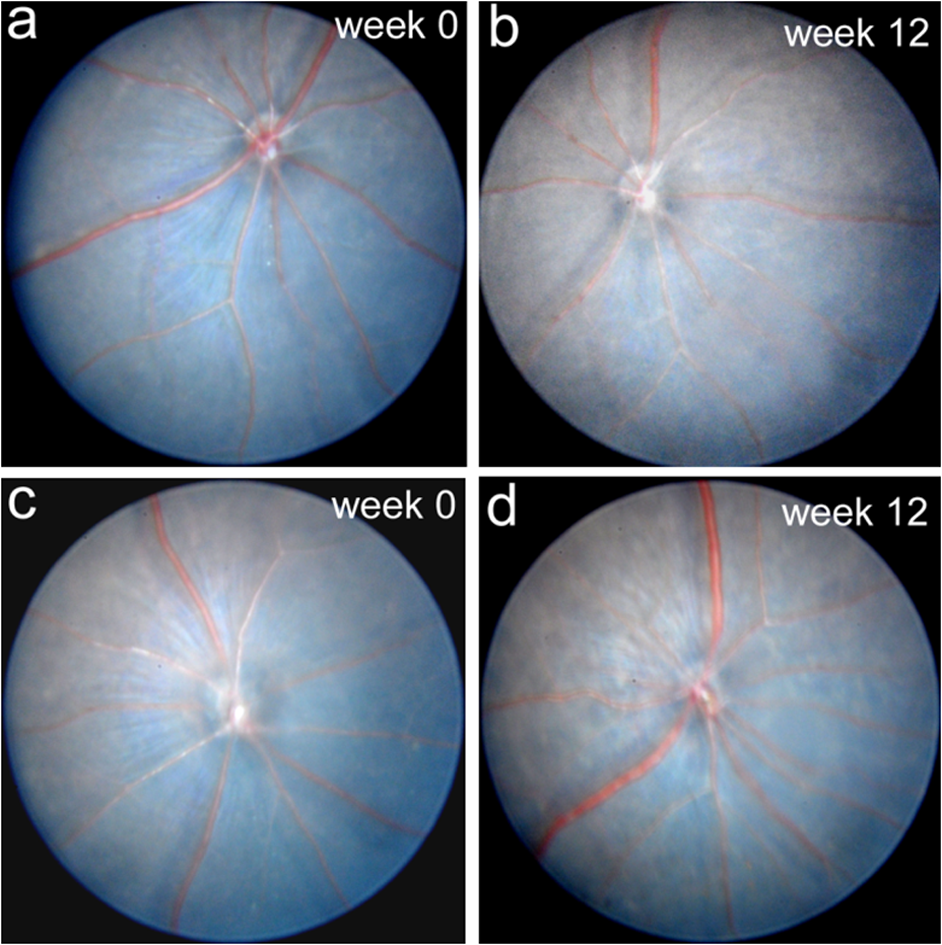


**Supplemental Figure 2. Retinal neuronal degeneration in Ins2^Akita^ mice following anti-VEGF treatment.** The thickness of each retinal layer was measured in retinal sections processed for GlyT1 (green channel), GABA (red channel) and DAPI (blue channel) following 5 intravitreal injections of anti-VEGF (a). (b) The average thickness of ONL. (c) The average thickness of OPL. (d) the average thickness of INL. (e), the average thickness of IPL. (f) The average thickness of GCL/NFL. n≥20 retinal images per strain/condition. Results are presented as mean ± SEM. *, P < 0.05; **, P < 0.01; ***, P < 0.001 compared to non-injected control group. One-way ANOVA. ONL, outer nuclear layer; OPL, outer plexiform layer; INL, inner nuclear layer; IPL, inner nuclear layer; GCL/NFL, ganglion cell layer/nerve fibre layer.


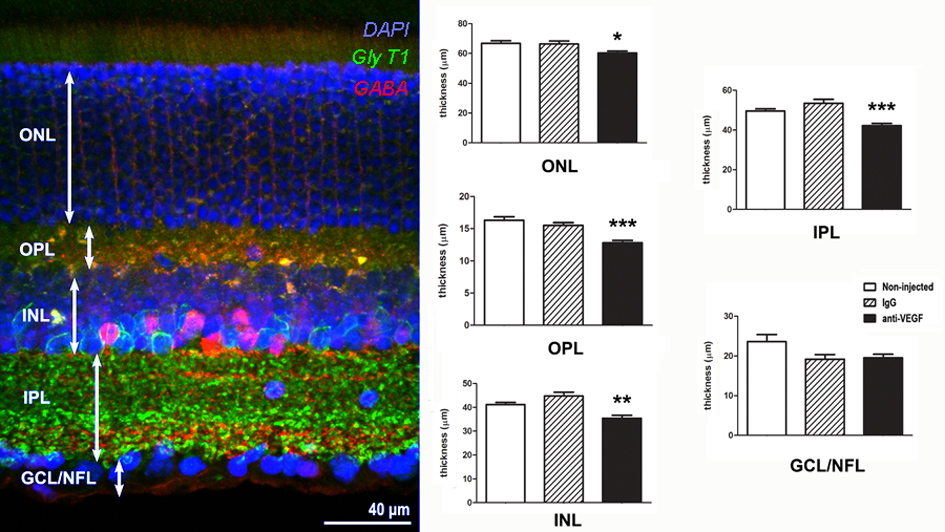


**Supplemental Figure 3. The effect of anti-VEGF treatment in retinal bipolar cells.** Retinal sections from Ins2^Akita^ mice after 5 intravitreal injections of anti-VEGF processed for secretagogin (a) or PKCα (b) immunoreactivity. (c) The density of cone bipolar cells (*box1)*, (d) rod bipolar cells (*box2*), (e) cone bipolar cell axonal boutons (thresholded black pixels enclosed in *box3*) and (f) rod bipolar cell axonal boutons (thresholded black pixels enclosed in *box4*). n≥20 retinal images per strain/condition. Results are presented as mean ± SEM. One-way ANOVA. INL, inner nuclear layer; IPL, inner nuclear layer.


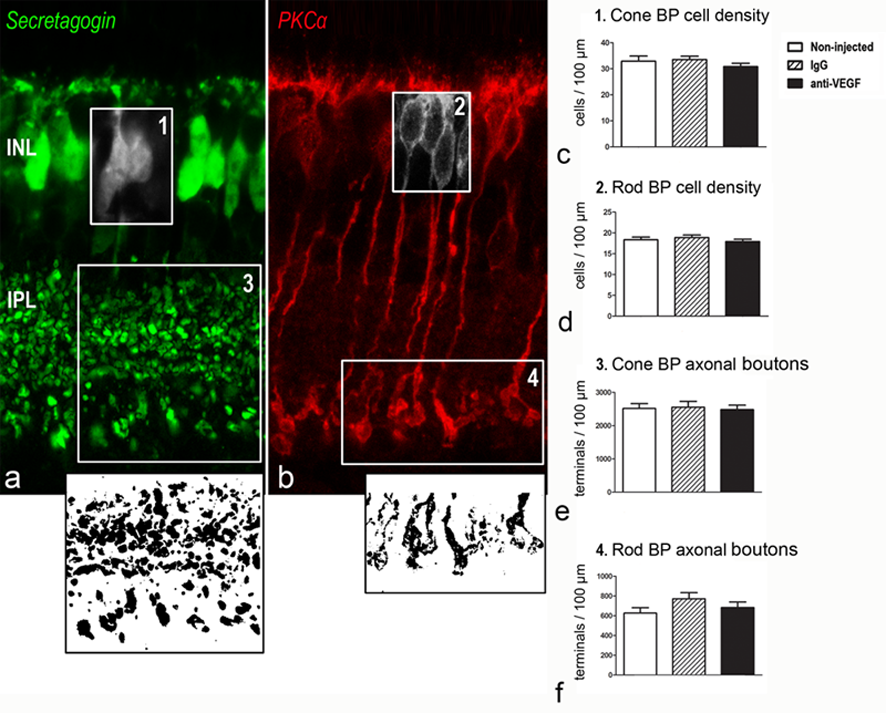


**Supplemental Figure 4. Diffusion of anti-VEGF and IgG antibodies in the neuroretina following intravitreal injection.** Retinal sections from non-injected WT mice (a) and 24 hours after intravitreal injection with IgG (b) or anti-VEGF (c) processed for Alexa fluor-488 donkey anti-goat IgG immunostaining. Diffusion of IgG and anti-VEGF through all retinal layers (arrowheads). ONL, outer nuclear layer; OPL, outer plexiform layer; INL, inner nuclear layer; IPL, inner nuclear layer; GCL/NFL, ganglion cell layer/nerve fibre layer.


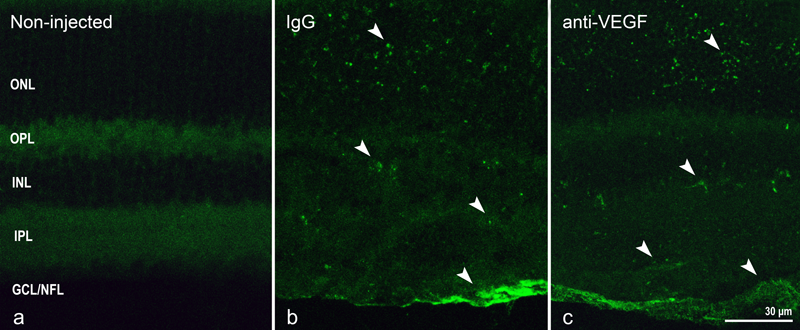

Supplement: Supplementary Information [file srep18316-s1.doc]
